# The Copper Metabolism MURR1 Domain Protein 1 (COMMD1) Modulates the Aggregation of Misfolded Protein Species in a Client-Specific Manner

**DOI:** 10.1371/journal.pone.0092408

**Published:** 2014-04-01

**Authors:** Willianne I. M. Vonk, Vaishali Kakkar, Paulina Bartuzi, Dick Jaarsma, Ruud Berger, Marten H. Hofker, Leo W. J. Klomp, Cisca Wijmenga, Harm H. Kampinga, Bart van de Sluis

**Affiliations:** 1 University Medical Center Utrecht, Department of Metabolic and Endocrine Diseases, and Netherlands Metabolomics Center, Utrecht, the Netherlands; 2 University Medical Center Utrecht, Complex Genetics Section, Utrecht, the Netherlands; 3 University of Groningen, University Medical Center Groningen, Department of Cell Biology, Groningen, the Netherlands; 4 University of Groningen, University Medical Center Groningen, Molecular Genetics, Groningen, the Netherlands; 5 Erasmus Medical Center, Department of Neuroscience, Rotterdam, the Netherlands; 6 University of Groningen, University Medical Center Groningen, Department of Genetics, Groningen, the Netherlands; Deutsches Zentrum für Neurodegenerative Erkrankungen e.V., Germany

## Abstract

The Copper Metabolism MURR1 domain protein 1 (COMMD1) is a protein involved in multiple cellular pathways, including copper homeostasis, NF-κB and hypoxia signalling. Acting as a scaffold protein, COMMD1 mediates the levels, stability and proteolysis of its substrates (e.g. the copper-transporters ATP7B and ATP7A, RELA and HIF-1α). Recently, we established an interaction between the Cu/Zn superoxide dismutase 1 (SOD1) and COMMD1, resulting in a decreased maturation and activation of SOD1. Mutations in *SOD1*, associated with the progressive neurodegenerative disorder Amyotrophic Lateral Sclerosis (ALS), cause misfolding and aggregation of the mutant SOD1 (mSOD1) protein. Here, we identify COMMD1 as a novel regulator of misfolded protein aggregation as it enhances the formation of mSOD1 aggregates upon binding. Interestingly, COMMD1 co-localizes to the sites of mSOD1 inclusions and forms high molecular weight complexes in the presence of mSOD1. The effect of COMMD1 on protein aggregation is client-specific as, in contrast to mSOD1, COMMD1 decreases the abundance of mutant Parkin inclusions, associated with Parkinson’s disease. Aggregation of a polyglutamine-expanded Huntingtin, causative of Huntington’s disease, appears unaltered by COMMD1. Altogether, this study offers new research directions to expand our current knowledge on the mechanisms underlying aggregation disease pathologies.

## Introduction

The ubiquitously expressed COMMD1 protein is the founder member of the *Co*pper *M*etabolism *M*URR1 *d*omain (COMMD) protein family. This class of proteins comprises ten members, and is highly conserved between eukaryotes and some protozoa [1]. Previously, COMMD1 was identified as a regulator of copper homeostasis as deletion of *COMMD1* resulted in an extensive accumulation of copper in the liver of Bedlington terriers and mice [2]. Various studies imply that COMMD1 controls copper homeostasis via an interaction with ATP7B and ATP7A, two copper-transporting proteins associated with the copper storage disorders Wilson’s and Menkes disease, respectively. Interestingly, whereas COMMD1 promotes the proteolysis of misfolded ATP7B proteins [3], the expression and subcellular localization of misfolded ATP7A mutants, and consequently their copper transporting function, are improved by COMMD1 [4].

Accumulating evidence indicates that COMMD1 is also involved in various other cellular pathways, including NF-κB and hypoxia signalling, sodium transport, and intracellular trafficking of membrane proteins such as the cystic fibrosis transmembrane conductance regulator (CFTR)[5]–[11]. As a scaffold protein, COMMD1 regulates the folding, stability, ubiquitination, and proteolysis of its interaction partners, including ATP7B and ATP7A, ENaC subunits, CFTR, RelA, and HIF-1α [5], [7], [9], [11]–[13]. Its chaperone-like function in the cellular protein homeostasis biology is underlined by its association with the ubiquitin-proteasome system (UPS).

Recently, we established the interaction between COMMD1 and the Cu/Zn Superoxide Dismutase 1 (SOD1), a ubiquitously expressed metalloenzyme involved in the cellular antioxidant defence [14]. The copper-dependent interaction between COMMD1 and SOD1 leads to a suppression of SOD1 homodimerization resulting in a marked decline in SOD1 scavenging activity and consequently an induction of toxic superoxide anions. At present, over 175 mutations in *SOD1,* primary associated with an autosomal dominant inheritance pattern (for updated list, see http://alsod.iop.kcl.ac.uk/), have been identified to cause the severe neurodegenerative disorder amyotrophic lateral sclerosis (ALS) [15]. In general, the dispersed mutations lead to misfolding and aggregation of the SOD1 protein [16]–[19], which may interfere with normal cellular functions [20]. Similar as mutant SOD1 (mSOD1), intraneuronal accumulation of misfolded Parkin and polyglutamine (polyQ)-expanded Huntingtin (Htt) proteins is associated with the neurodegenerative disorders Parkinson’s and Huntington’s disease, respectively [21]–[26]. Although the processes underlying these aggregation disease pathologies might share the gain-of-toxic aggregation as mechanism of toxicity, the different misfolded proteins form biochemically distinct aggregates [27], and affect different types of neurons or neuronal areas (i.e. dopaminergic neurons in substantia nigra of midbrain (Parkinson’s disease), medium spiny neurons in striatum and frontal and temporal cortices (Huntington’s disease), or motor neurons in spinal cord and brain stem (ALS)), thus resulting in distinctive clinical phenotypes of these aggregation diseases.

Based on the established interaction of COMMD1 with SOD1, and the present studies implicating a general role for COMMD1 in the protein quality control of various (misfolded) clients, we assessed the effect of COMMD1 on the aggregation of the aggregation-prone mutant SOD1, Parkin and Htt proteins. Here, we show that COMMD1 co-localizes to and increases the formation of mSOD1 aggregates. On the contrary, COMMD1 reduces the abundance of mutant Parkin C289G high molecular weight (HMW) species, whilst it has no significant effect on polyglutamine (polyQ)-expanded Htt aggregation.

## Materials and Methods

### Reagents

The following antibodies were used for immunoblotting: monoclonal mouse-anti-Flag M2 and polyclonal mouse-anti-Flag M2 HRP-conjugated (Sigma-Aldrich, St. Louis, MO, USA), polyclonal rabbit-anti-GST (Santa Cruz Biotechnology, Santa Cruz, CA, USA), polyclonal mouse-anti-HA (Sigma-Aldrich), rabbit-anti-COMMD1 antiserum [28], monoclonal mouse-anti-GFP JL-8 (Clontech, Mountain View, CA, USA), monoclonal mouse-anti-Parkin (Park8) (Cell Signaling, Danvers, MA, USA), monoclonal mouse-anti-GAPDH (RDI Research Diagnostics, Concord, MA, USA), and polyclonal mouse-anti-α-Tubulin (Sigma-Aldrich).

### Constructs

pEBB-HA-COMMD1, pEBB-COMMD1-Flag, mCherry-COMMD1, COMMD1-GST, and pEBB-SOD1-Flag constructs were described previously [14], [29]. eGFP-N2 construct was commercially obtained from Invitrogen Life Technologies Corporation (Carlsbad, CA, USA). Mutations in pEBB-SOD1-Flag WT were introduced by the Quickchange site-directed mutagenesis method (Stratagene, primer sequences are listed in Table S1). pcDNA3.1-SOD1-YFP WT, G85R and G93A plasmids were obtained from Dr. J. Frydman (Stanford, CA, USA). The mutations were verified by automated DNA sequence analysis. eYFP-HttQ74 encoding for the eYFP-tagged Huntingtin N-terminal fragment of exon 1 with 74 CAG repeats was kindly provided by Dr. D. C. Rubinsztein [30]. eYFP-HttQ119 and DNAJB6 constructs were described previously [31]. The pcDNA3.1-Flag-Parkin WT and C289G constructs were used as described previously [32], and kindly provided by Dr. M. Cheetham (UK).

### Cell culture and transfections

Human embryonic kidney 293T (HEK293T), HeLa and mouse neuroblastoma Neuro2A cells (all obtained from ATCC, Manassas, VA, USA) were cultured in high-glucose Dulbecco’s modified Eagle’s medium GlutaMAX-TM (4.5 g/L D-Glucose and Pyruvate; Invitrogen Life Technologies Corporation) supplemented with 10% fetal bovine serum (FBS), L-glutamine, penicillin and streptomycin at 37°C in 5% CO_2_. Monoclonal HEK293T cell lines, stably transfected with pSUPER-RETRO vector (shControl) or a plasmid encoding short hairpin RNA (shRNA) targeting COMMD1 mRNA sequence (shCOMMD1), were described previously [33] and maintained in HEK293T medium supplemented with 1 μg/μl puromycin dihydrochloride (Sigma-Aldrich). For copper-dependent interaction studies, cells were incubated overnight with 150 μM CuCl_2_ prior to lysis.

Cells were transiently transfected by the calcium phosphate precipitation method or FUGENE HD transfection method (Roche, Basel, Switzerland), according to manufacturer’s instructions. Unless stated otherwise, cells were harvested 48 h after transfection for biochemical analysis.

### GST pull down assays and immunoblot analyses

Precipitation of GST-tagged proteins by means of GSH-sepharose beads was performed as described previously [14]. In short, transiently transfected cells were rinsed once with PBS prior to lysis in lysisbuffer A (25 mM HEPES; pH 7.9, 100 mM NaCl, 1 mM NaEDTA, 1% Triton X-100, 10% Glycerol) supplemented with 1 mM Na_3_VO_4_, 1 mM PMSF, 10 mM DTT and protease inhibitors (Complete; Roche). For lysis of copper-treated cells, lysis buffer A was supplemented with 1 mM CuCl_2_. Protein concentrations were determined by Bio-Rad (Bradford) Protein Assay (Bio-Rad Laboratories Inc., Hercules, CA, USA). In all interaction studies, equal amounts of proteins in lysisbuffer A were used for precipitation. Protein lysates were boiled at 95°C prior to gel loading, and SDS–PAGE was followed by proteins transfer onto nitrocellulose membranes (Schleicher & Schuell,‘s-Hertogenbosch, the Netherlands) for immunoblot analysis. Input samples represent approximately 1% of protein amounts used for GST-precipitation.

### Visualization of protein multimeric complexes and aggregates

Visualization of SOD1 monomers, dimers and multimers by means of non-reducing SDS-PAGE was performed as described previously [14]. Aggregates of SOD1 mutant proteins were visualized by means of filter trap analysis as described by Niwa *et al*. [34] with slight modifications. In short, cells were lysed in lysisbuffer B (50 mM Tris-HCL, pH 8.0; 150 mM NaCl; 1 mM EDTA and 1% NP-40) supplemented with protease inhibitors (Complete; Roche). After centrifugation, supernatant was separated from pellet, and the pellet was dissolved in Buffer C (50 mM Tris-HCL, pH 8.0; 1 mM MgCl_2_) supplemented with ∼250 U Benzonase endonuclease (Merck Chemicals, Darmstadt, Germany) for 1 h at 37°C. To complete pellet dissolvement, samples were subsequently incubated with Buffer D (Buffer B supplemented with 2% SDS) on a shaking platform for 1 h prior to addition of Buffer E (40 mM EDTA supplemented with 4% SDS). 40 μg protein lysate was applied on 0.2 μm cellulose acetate membranes, prewashed with TBS-0.1% SDS, following detection of proteins by means of immunoblotting. Blue Native PAGE experiments were performed using precast NativePAGE Novex 4–16% Bis-Tris Gels (Invitrogen Life Technologies Corporation), according to manufacturer’s guidelines. For polyQ experiments, filter trap assay was used to assess HMW species as described previously [31]. PolyQ and Parkin samples were lysed after 24 h in 1% (v/v) Triton X-100 in PBS containing 1% (v/v) proteinase inhibitor cocktail for 15 min on ice. Cell lysates were scraped and centrifuged at 13.000 g for 15 min at 4°C. The supernatant was used as soluble fraction and the pellet was resuspended in 1% SDS buffer, sonicated, and used as insoluble or pellet fraction.

### Confocal Microscopy

For immunofluorescence, cells were grown on coverslips pre-coated with poly-L-lysine (Sigma-Aldrich) prior to washing the cells once in ice-cold PBS and fixation using 4% (w/v) paraformaldehyde for 20 min at 4°C 72 h post-transfection. Subsequently, coverslips were mounted using Immu-Mount (Thermo Fisher Scientific, Houston, TX, USA). Images were acquired using a LSM510 Meta confocal microscope (Carl Zeiss, Jena, Germany) equipped with a 63x/1.40 N.A. Plan-Apochromat oil-objective by sequential excitation at 488 and 561 nm.

### Statistical analysis

**Figure 1:**
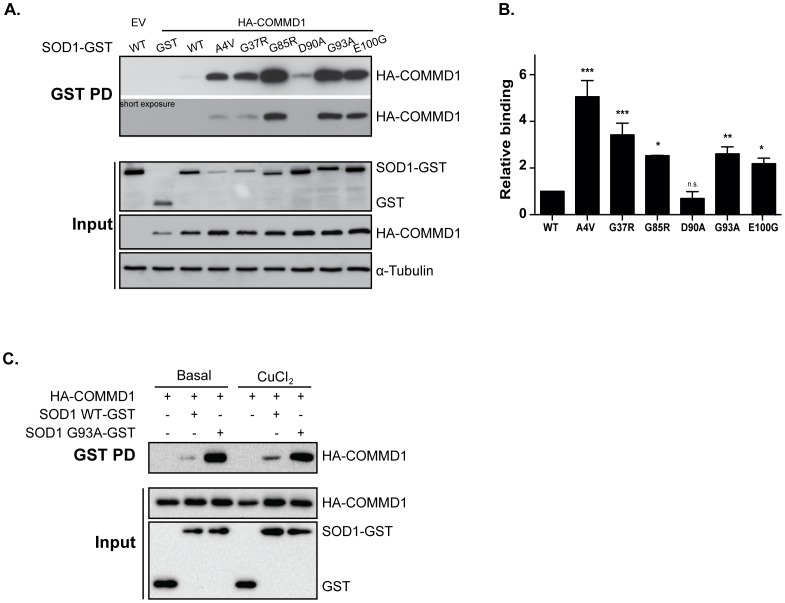
Enhanced interaction of COMMD1 with ALS-associated SOD1 mutant proteins relative to SOD1 wild-type. **A.** HEK293T cells were transient transfected with empty vector (EV), HA-COMMD1 alone or in combination with SOD1-GST constructs (WT, A4V, G37R, G85R, D90A, G93A, E100G). GST-proteins were precipitated by means of GSH-sepharose beads prior to detection of their interaction with COMMD1 as visualized by immunoblotting for HA-COMMD1 (GST PD; upper panel), as described previously [27]. 30 μg of protein lysates were used for detection of WT and mutant SOD1-GST and HA-COMMD1 in total cell lysates (Input; lower panel) using antibodies directed against the HA- or GST-fusion proteins. Tubulin was used as loading control. **B.** Densitometric quantification of interaction strength between COMMD1 and SOD1 WT versus SOD1 mutants (A4V, G37R, G85R, D90A, G93A, E100G; GST PD), normalized for total SOD1 expression (input). Binding of COMMD1 to SOD1 WT was set at 1. * indicates significantly increased binding of COMMD1 to mSOD1 compared to SOD1 WT – COMMD1 (* p<0.05, ** p<0.005, *** p<0.0001). n.s.  =  non-significant. **C.** HEK293T cells were transient transfected with HA-COMMD1 alone or in combination with SOD1-GST constructs (WT and G93A). Cells were incubated overnight under basal conditions or with 150 μM CuCl_2,_ lysed, and GST fusion proteins were precipitated by means of GSH-sepharose beads prior to detection of their interaction with COMMD1 as visualized by immunoblotting for HA-COMMD1 (GST PD; upper panel). 30 μg of protein lysates were used for detection of SOD1-GST WT and G93A and HA-COMMD1 in total cell lysates (Input; lower panel) using antibodies directed against the HA- or GST-tags.

Relative protein expressions and interaction strengths were quantified using densitometry (GS-700; Bio-Rad). The quantitative data in this paper is represented as means ± SD. Statistical evaluation was made using a paired One-way ANOVA with Dunnett’s multiple comparison test (Figure 1B) and paired Student’s t-test. Differences we­re considered to be significant at p<0.05.

## Results

### Enhanced interaction of COMMD1 with ALS-associated SOD1 mutants compared to SOD1 wild-type

To assess a potential effect of altered COMMD1 expression for the fate of mutant SOD1, we first examined the interaction of COMMD1 with a range of mSOD1 proteins associated with ALS (A4V, G37R, G85R, D90A, G93A and E100G) by means of GST pull down studies in HEK293T cells. The SOD1 mutants studied were selected as they are located in distinct regions of the SOD1 protein, and associated with an ALS disease pathology in transgenic rodent models [35]–[40]. With the exception of mSOD1 D90A, a significant increase in COMMD1 binding to the SOD1 mutants compared to SOD1 WT was observed (Figure 1A). Quantification of the interactions, which was corrected for SOD1 input levels, demonstrated that the binding of the mSOD1 proteins to COMMD1 was up to a 5-fold enhanced compared to SOD1 WT (Figure 1B). To assess whether the enhanced interaction of COMMD1 with SOD1 mutants was not due to any potential sedimentation of the aggregating mSOD1 proteins during low speed centrifugation, we confirmed the increased COMMD1-mSOD1 association in additional GST pull down studies using glutathione magnetic beads (Figure S1A). Consistent with the precipitation of mSOD1-GST proteins, the enhanced binding of COMMD1 to mSOD1 was established bidirectionally as co-immunoprecipitation of mSOD1-Flag by COMMD1-GST using glutathione magnetic beads also clearly identified the COMMD1-(m)SOD1 interaction (Figure S1B). In contrast to its interaction with SOD1 WT [14] and mSOD1 D90A, the binding of COMMD1 to all studied mSOD1 proteins occurred independently of copper (Figure 1C, S1C, and data not shown).

### COMMD1 modulates the aggregation of SOD1 mutants

**Figure 2:**
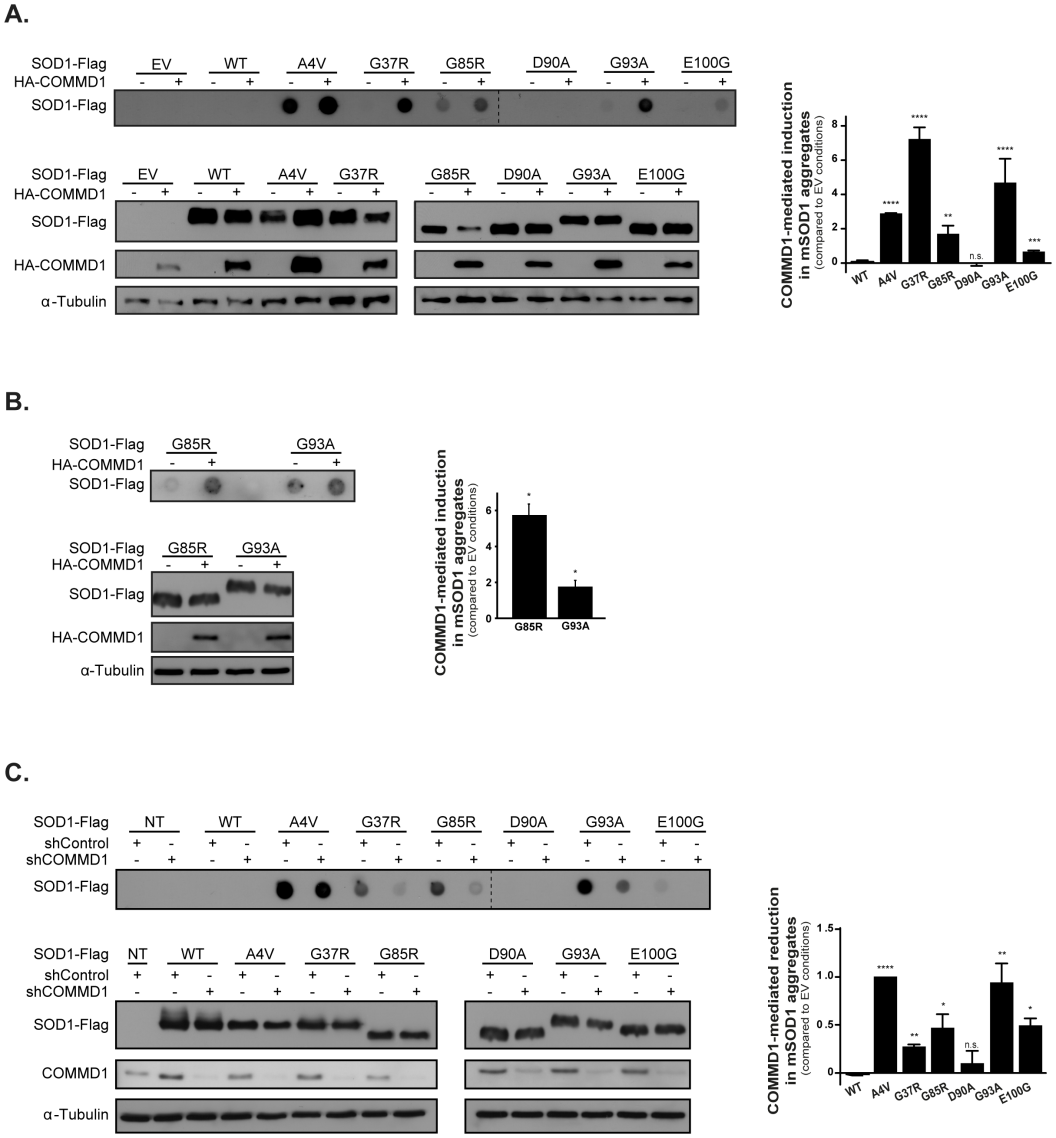
COMMD1 mediates aggregation of ALS-associated SOD1 mutants. **A.** SOD1-Flag WT and mutant (A4V, G37R, G85R, D90A, G93A, E100G) constructs were co-transfected with either EV (–) or HA-COMMD1 in HEK293T cells prior to detection of SOD1 aggregates by means of filter trap analysis on cellulose acetate membrane as described in Experimental procedures section (40 μg protein; upper panel). Aggregates were analysed on the same membranes and visualized by IB using antibodies directed against the Flag-tag. Input samples (15 μg protein; lower panel), corresponding to the filter trap analysis, represent total cell lysates of each condition, and were visualized using antibodies directed against the Flag- and HA-tag, and α-Tubulin, respectively. Input samples were analysed on separated membranes. Graph represents the relative COMMD1-mediated induction in SOD1 aggregates compared to EV conditions (representative for at least 3 independent experiments). EV, SOD1 WT and mSOD1 D90A values were considered as 0 as these proteins do not form aggregates. * indicates significantly increase in mSOD1 aggregation mediated by COMMD1 (** p<0.005, *** p<0.001, **** p<0.0001). n.s.  =  non-significant. **B.** Filter trap analysis of Neuro2A cells transiently transfected with SOD1-Flag mutants G85R and G93A in combination with either EV (–) or HA-COMMD1. Detection of aggregates and input samples was performed as described in Figure 2A. Graph represents the relative COMMD1-mediated induction in mSOD1 aggregates compared to EV conditions (representative for at least 3 independent experiments). * indicates significantly increase in mSOD1 aggregation mediated by COMMD1 (* p<0.05). **C.** Filter trap analysis performed in HEK293T cells stably transfected with shControl or shCOMMD1 and co-transfected with SOD1-Flag WT and mutants (A4V, G37R, G85R, D90A, G93A, E100G). Aggregates were analysed on the same membranes. Analyses were performed as described in Figure 2A. Input samples were analysed on separated membranes. Indicated values represent the relative COMMD1-mediated reduction in mSOD1 aggregates compared to EV conditions (mSOD1 aggregation upon EV co-transfection was set a 1.0; representative for at least 3 independent experiments). EV, SOD1 WT and mSOD1 D90A values were considered as 0 as these proteins do not form aggregates. Graph represents the relative COMMD1-mediated reduction in SOD1 aggregates compared to EV conditions (representative for at least 3 independent experiments). EV, SOD1 WT and mSOD1 D90A values were considered as 0 as these proteins do not form aggregates. * indicates significantly decrease in mSOD1 aggregation upon COMMD1 knock-down (* p<0.05, ** p<0.005, **** p<0.0001). n.s.  =  non-significant.

Since mSOD1 proteins are susceptible to aggregate formation, a pathological hallmark of ALS, we determined whether COMMD1 expression affects mSOD1 the formation of SDS-insoluble aggregates by means of filter trap analysis. In contrast to SOD1 WT and mSOD1 D90A, the mSOD1 proteins formed SDS-insoluble aggregates in HEK293T cells. Co-expression of COMMD1 significantly increased the formation of these SDS-insoluble mSOD1 aggregates (Figure 2A; upper panel). To rule out the possibility that the effect of COMMD1 on mSOD1 aggregation is due to elevated expression of mSOD1, we analysed the total mSOD1 protein levels by Western blot analysis. Except for the A4V mutant, which showed a slight increase upon COMMD1 co-transfection, all other mutants were either unaffected or even showed reduced expression in the presence of COMMD1 (Figure 2A; lower panel). Similar to the effect in HEK293T cells, COMMD1 also augmented mSOD1 aggregation in the neuroblastoma cell line Neuro2A, as demonstrated by mutants G85R and G93A (Figure 2B). The inverse was observed upon reduction of the endogenous COMMD1 expression in HEK293T cells using shRNA, which significantly suppressed the formation of mSOD1 aggregates without affecting mSOD1 levels (Figure 2C).

### COMMD1 promotes the formation of multimeric mSOD1 complexes

**Figure 3:**
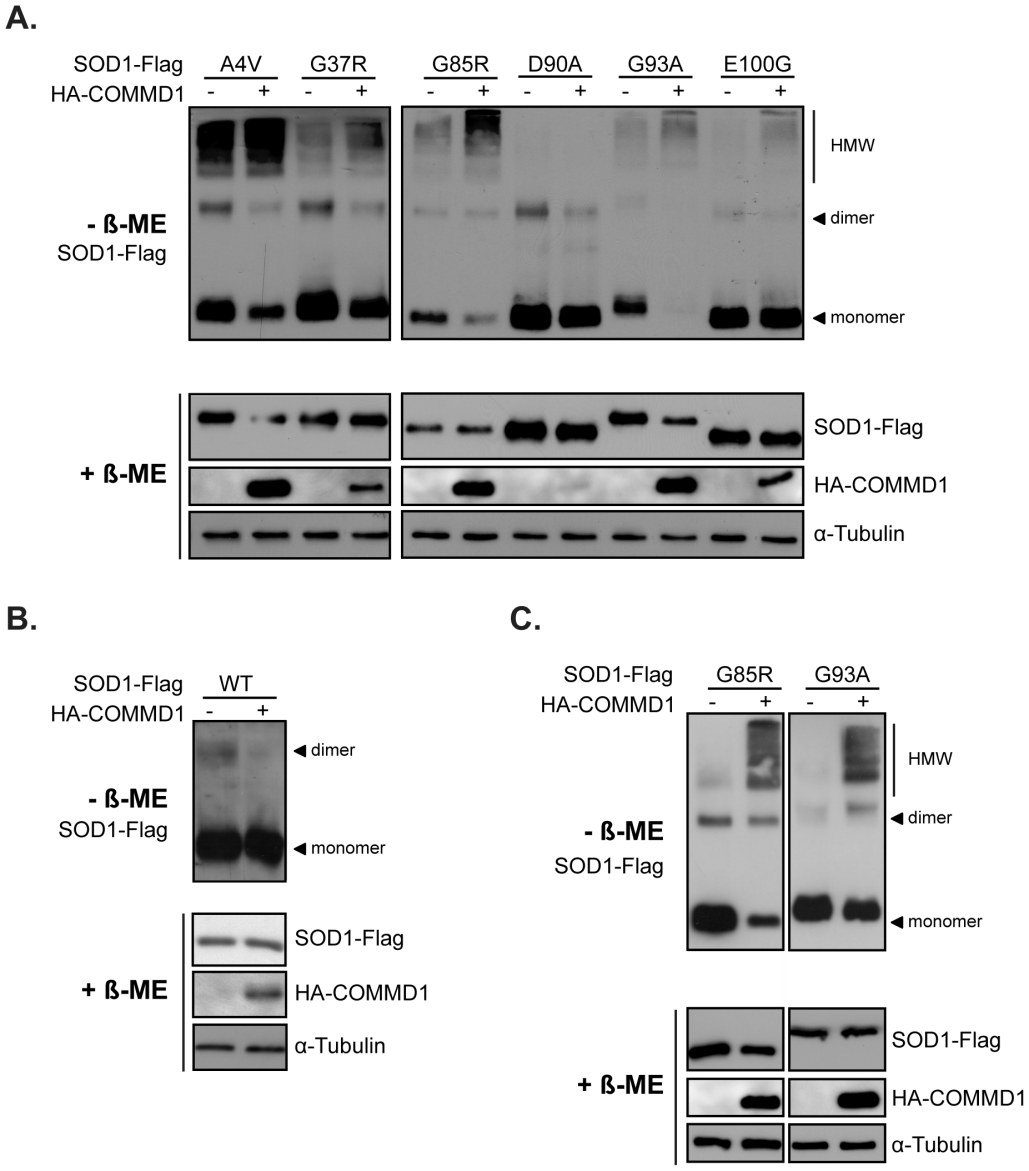
Formation of mSOD1 high molecular mass species is regulated by COMMD1. **A.** HEK293T cells were co-transfected with SOD1-Flag mutants (A4V, G37R, G85R, D90A, G93A, E100G) in combination with EV (–) or HA-COMMD1 prior to lysis and detection of SOD1 multimeric complexes using SDS-PAGE under non-reducing conditions (– β-ME; 40 μg). Immunoblotting was performed using antibodies directed against the Flag-tag and HA-COMMD1 proteins. Input samples were analysed in presence of β-ME (+ β-ME; 30 μg). Samples were analysed on separated membranes. **B.** HEK293T cells were co-transfected with SOD1-Flag WT in combination with EV (–) or HA-COMMD1 prior to lysis and analysed as described in Figure 3A. **C.** Visualization of SOD1 HMW complexes in Neuro2A cells transfected with SOD1-Flag mutants G85R and G93A in combination with EV (–) or HA-COMMD1 as described in Figure 3A. Samples were analysed on separated membranes.

Next, we investigated whether COMMD1 also affects SOD1 dimerization and the formation of multimeric mSOD1 complexes using non-reducing SDS-PAGE analysis. In line with the filter trap assay, the majority of the mSOD1 proteins studied formed extensive amounts of HMW complexes in HEK293T cells, which were markedly increased by expression of COMMD1. This occurred at the expense of loss of the dimeric and monomeric forms of SOD1 (Figure 3A; – β-ME); again, this was not due to an increase in mSOD1 expression as in fact the total protein levels of most of the mutants were reduced (Figure 3A; + β-ME). Of note, in presence of the reducing agent (+β-ME), no mSOD1 HMW species were detected at the top, nor in the stacking, of the SDS-PAGE gels, indicating that all mSOD1 HMW species are concentrated in the monomeric form (data not shown). Mutant D90A did not show substantial HMW complexes and these were also not induced by COMMD1. However, similar to what we previously have demonstrated for SOD1 WT ([14] and Figure 3B), COMMD1 reduced the formation of mSOD1 D90A homodimers (Figure 3A). Analogous to HEK293T cells, exogenous expression of COMMD1 also resulted in an increase of multimeric mSOD1 complexes in Neuro2A cells as illustrated by the SOD1 mutants G85R and G93A (Figure 3C).

**Figure 4:**
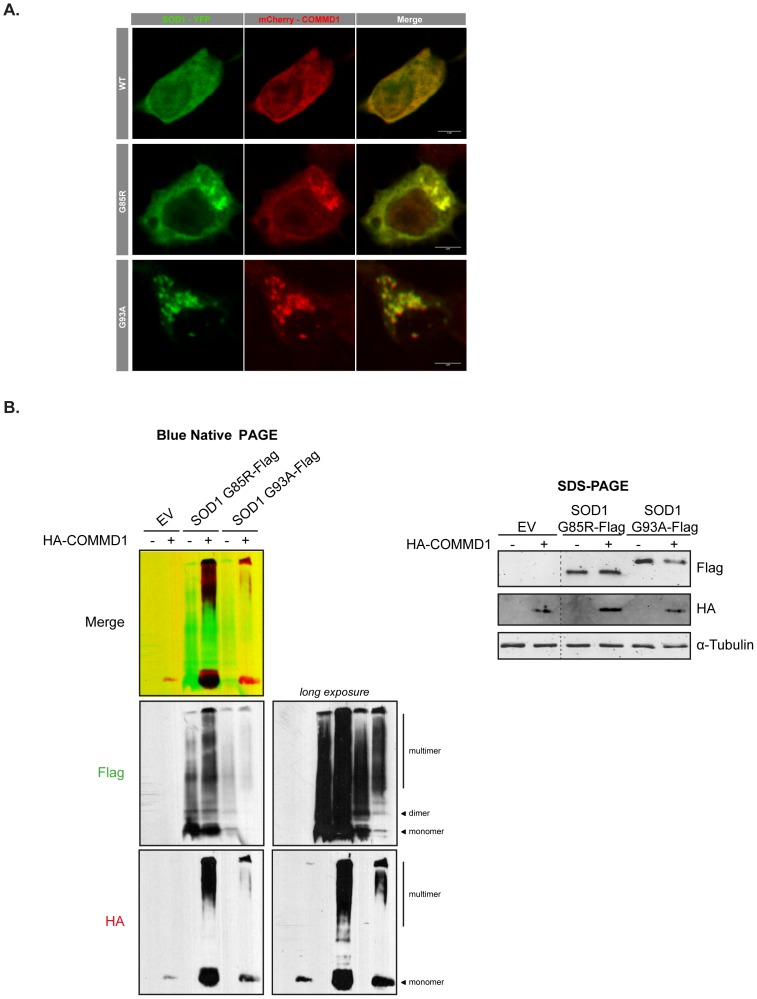
COMMD1 is in complex with aggregated mSOD1 proteins. **A.** Neuro2A cells were transient transfected with eYFP-tagged SOD1 WT, or mSOD1 G85R and G93A, with or without mCherry-COMMD1, as indicated. 72 h post-transfection, cells were fixed and imaged using a confocal microscope. **B**. HEK293T cells were transient transfected with either EV (–) or HA-COMMD1 in combination with EV, or Flag-tagged mSOD1 G85R or G93A constructs prior to lysis and detection of SOD1 multimeric complexes using blue native PAGE (30 μg). Immunoblotting was performed using antibodies directed against the Flag-tag and HA-COMMD1 proteins. Total protein levels were analysed in presence of β-ME on a SDS-PAGE gel (30 μg). Photoshop analysis was used to generate overlay, using a marked region on the membrane as focus point.

Interestingly, COMMD1 co-localizes to mSOD1 inclusions using confocal microscopy analysis (Figure 4A), while mCherry-COMMD1 is not sequestered into puncta in cells expressing SOD1 WT proteins (Figure 4A). This observation is strengthened by the fact that COMMD1 co-migrates with multimeric mSOD1 on a blue native PAGE gel, whereas, in absence of mSOD1, COMMD1 is only present as a monomeric protein as COMMD1 HMW species remain undetectable (Figure 4B). Together, these observations may suggest that COMMD1 has to be in complex with multimeric mSOD1 to accelerate mSOD1 aggregation.

### COMMD1-mediated mSOD1 aggregation is dependent on SOD1 Cys6

**Figure 5:**
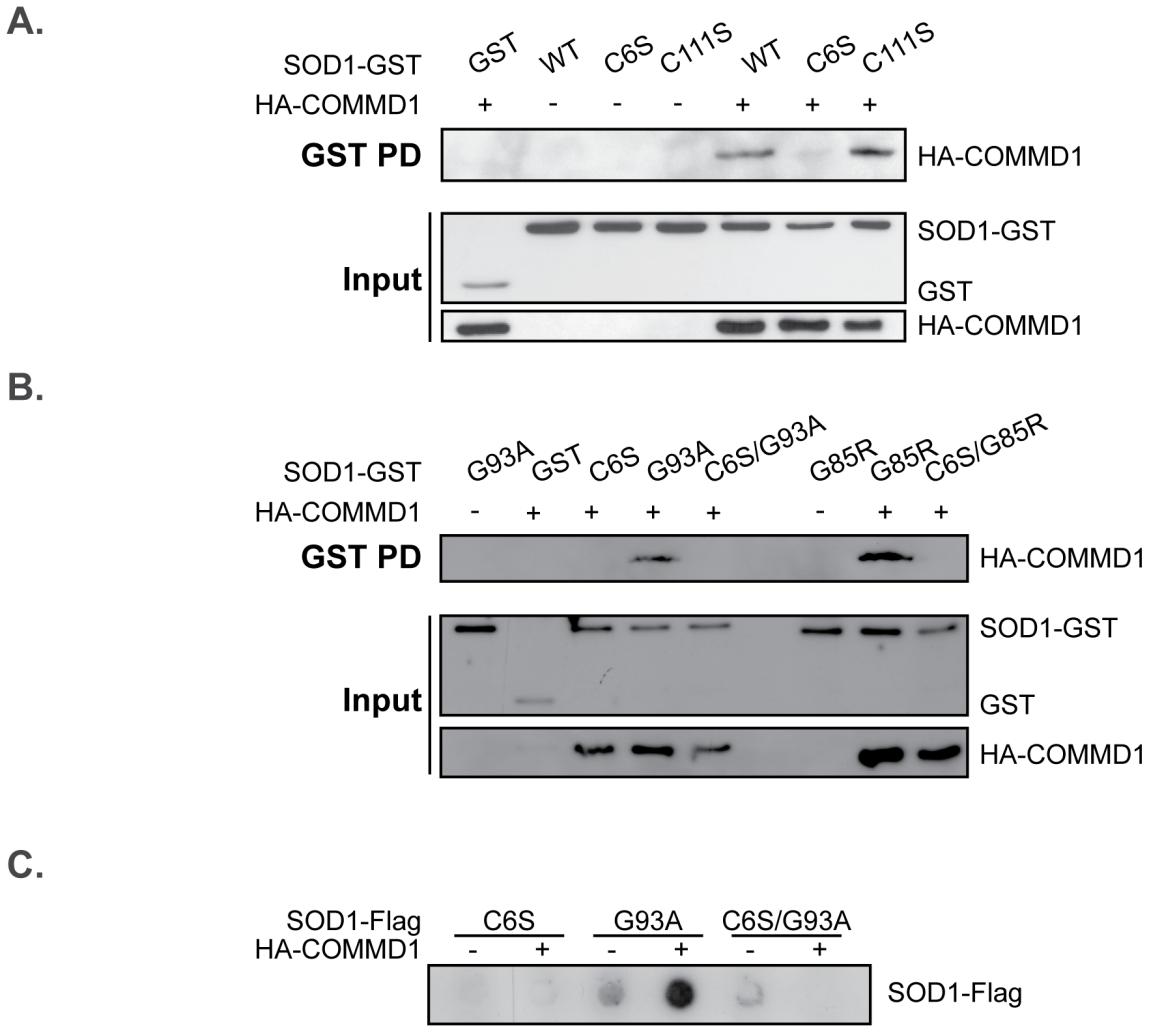
COMMD1-mediated induction of mSOD1 aggregation is affected by a SOD1 C6S substitution. **A.** HEK293T cells were transient transfected with either EV (–) or HA-COMMD1 in combination with GST only or SOD1-GST constructs (WT, C6S, C111S). GST PD was performed as described under Figure 1A. **B.** HEK293T cells were transient transfected with EV (–) or HA-COMMD1 in combination with GST only or SOD1-GST constructs (WT, C6S, G93A, C6S/G93A, G85R, C6S/G85R). GST PD was performed as described under Figure 1A. **C.** Filter trap analysis of HEK293T cells transiently transfected with SOD1-Flag mutants (C6S, G93A, C6S/G93A) in combination with either EV (–) or HA-COMMD1. Detection of aggregates was performed as described in Figure 2A.

Previous studies are indicative for detergent-insoluble mSOD1 aggregates to contain molecules that are cross-linked by intermolecular disulfide bonds, and that these promote the formation of the mSOD1 HMW complexes [34], [41]–[43]. However, these data have recently been debated by other studies that only show appearance of aberrant disulfide linkages in a late-disease state [44]–[46]. SOD1 contains four cysteine residues. Whereas the cysteines at position 57 and 146 form an intramolecular disulfide bond within the SOD1 monomer, the cysteines at position 6 and 111 can form intermolecular disulfide bonds and are suggested to be involved in mSOD1 oligomer assembly [34], [47]. To examine whether intermolecular disulfide bond formation is associated with the enhanced SOD1 aggregation in the presence of COMMD1, cysteines 6 and 111 in the SOD1 sequence were mutated into a serine (SOD1 C6S and C111S, respectively), and their effect on the SOD1 - COMMD1 interaction and COMMD1-mediated aggregation was established. Interestingly, binding of COMMD1 to SOD1, both WT and ALS-linked mutants, was almost completely abolished by SOD1 C6S, whereas it remained unaltered upon mutating the cysteine at position 111 (Figure 5A (SOD1 WT) and B (mSOD1), and data not shown). Previously, this C6S mutation drastically minimized the ability of mSOD1 G85R and G93A to form disulfide-linked HMW complexes, whereas the effect of the C111S substitution in these proteins was much less pronounced [34], [48]. This, together with the knowledge that the double SOD1 mutant C6S/G93A does not display any aggregates, neither in the presence of exogenous COMMD1 ([34] and Figure 5C), not only suggest that cysteine residue 6 of SOD1 is essential for COMMD1 – mSOD1 interaction, but also for the formation of mSOD1 aggregates. Noteworthy, an amino acid substitution of the single cysteine present in the COMMD1 sequence into a serine (COMMD1 C160S) had no clear effect on mSOD1 aggregation (data not shown), implying that the increased aggregation of mSOD1 by COMMD1 is unlikely resulting from intermolecular disulfide linkages.

### COMMD1 mediates aggregate formation in a client-specific manner

**Figure 6:**
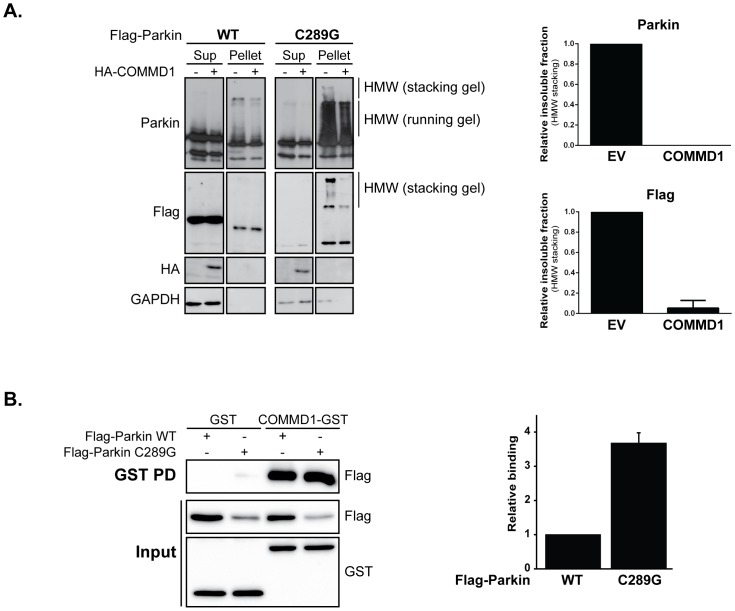
COMMD1 diminishes Parkin C289G aggregation. **A.** HEK293T cells were transfected with Parkin WT and C289G constructs alone, or in combination with HA-COMMD1. Supernatant (Sup) and pellet fractions were prepared as described in Experimental procedures. Immunoblotting was performed using indicated antibodies. Graphs represent relative fraction of insoluble Parkin proteins in absence or presence of exogenous COMMD1 (fractions quantified from both Parkin as well as Flag immunoblots). **B.** HEK293T cells were transient transfected with GST or COMMD1-GST alone in combination with Flag-Parkin constructs (WT and C289G). Cells were lysed, and GST fusion proteins were precipitated as described under Figure 1A. Immunoblotting was performed using indicated antibodies. Densitometric quantification of interaction strength between COMMD1 and Parkin WT versus C289G mutant, normalized for total Parkin expression (input). Binding of COMMD1 to Parkin WT was set at 1.

Next, we assessed whether COMMD1 also affects the aggregate deposition of other aggregation-prone misfolded proteins, such as a Parkin RING domain mutant (C289G) associated with Parkinson’s disease. Consistent with previous findings, the fraction of Parkin ending up in the Triton-insoluble pellet fraction is dramatically enhanced upon this cysteine to glycine substitution at position 289 as detected by SDS-PAGE; in this insoluble fraction, there is an increase in Parkin-containing HMW species, all indicative of aggregation ([25] and Figure 6A; pellet fraction). In contract to what we found for mSOD1, ectopically expressed COMMD1 suppressed Parkin C289G aggregation (Figure 6A; pellet fraction). Using a pull down assay, a clear interaction between COMMD1 and Parkin WT as well as Parkin C289G was detected; yet, the mutation in Parkin increased its binding to COMMD1 (Figures 6B (GSH sepharose beads) and S2 (GSH magnetic beads)), similar to what was seen for the SOD1 mutants studied (Figure 1).

**Figure 7:**
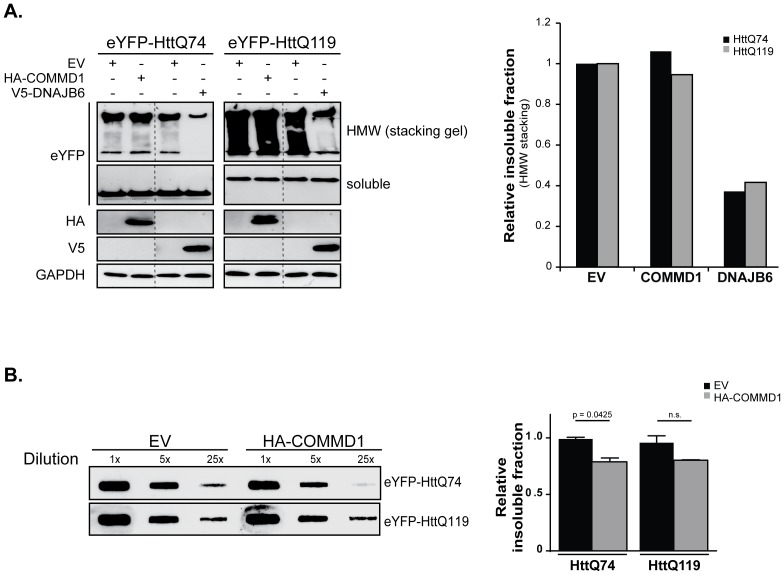
Aggregation of polyglutamine proteins is unaffected by COMMD1. **A.** HEK293T cells were transfected with eYFP-HttQ74 and eYFP-HttQ119 alone, or in co-expression with HA-COMMD1 or V5-DNAJB6 prior to lysis and detection of polyQ HMW complexes using SDS-PAGE. HMW aggregating complexes were trapped and detected in the stacking gel. IB was performed using indicated antibodies. Samples were analysed on the same membranes. Graph represents the relative fraction of insoluble HttQ74 and Q119 HMW species under EV conditions or upon co-expression of COMMD1 or DNAJB6. Insoluble Htt HMW species present in EV conditions were set at 1. **B.** Filter trap analysis of HEK293T cells transfected with the polyQ proteins eYFP-HttQ74 and eYFP-HttQ119, in co-expression with EV or HA-COMMD1. Samples were prepared as described under Experimental procedures and diluted as indicated. Immunoblotting was performed antibodies directed against the eYFP-tag. Densitometric quantification of insoluble fraction of HttQ74 and HttQ119, in absence or presence of exogenous COMMD1 (5x dilution). EV conditions were set at 1. p  =  0.0425, indicates a significant decrease in insoluble HttQ74 aggregates mediated by COMMD1. n.s.  =  non-significant.

In contrast to mutant SOD1 and Parkin aggregates, COMMD1 has no clear effect on the aggregation of a fragment encoding the N-terminal region of exon 1 of the *Huntingtin* gene with either 74 or 119 glutamine repeats (HttQ74 and HttQ119) associated with Huntington’s disease. Consistent with the inability to identify an interaction between COMMD1 and HttQ74 or HttQ119 proteins (data not shown), no changes in the Htt HMW aggregating species could be identified by Western blotting (HMW are trapped in the stacking gel; Figure 7A), whilst, as a positive control, enhanced expression of the DNAJ (Hsp40 homolog) chaperone family protein DNAJB6 clearly reduced the polyQ aggregation (Figure 7A and [31]). A slight reduction in SDS-insoluble Htt aggregates in cells expressing COMMD1 was determined though in a filter retardation assay (Figure 7B). However, since COMMD1 does not interact with Htt proteins and its effect on Htt aggregate formation is only very minor if any, we believe that it is unlikely for COMMD1 to be a modulator of polyQ-expanded Htt aggregation.

Together, these data imply that COMMD1 affects the aggregation of the three tested disease-associated misfolded proteins in a client-specific manner.

## Discussion

Recent studies demonstrated that COMMD1 is involved in different cellular processes, such as copper metabolism, sodium transport, and NF-κB and HIF signalling. Here, we show a client-specific role for COMMD1 on the aggregation of protein species associated with the neurodegenerative disorders SOD1-linked ALS, Parkinson’s disease and Huntington’s disease. Down regulation of COMMD1 expression suppresses the deposition of mSOD1, whereas exogenous COMMD1 exacerbates the abundance of mSOD1 aggregates. Inversely, COMMD1 decreases the aggregation of Parkin C289G, while aggregation associated with the expression of polyQ-expanded Huntingtin was unaffected by COMMD1.

Although the mechanism by which COMMD1 exerts its effect on the aggregation-prone proteins needs further investigation, our data suggest that the COMMD1-induced mSOD1 aggregation is regulated by a physical interaction between COMMD1 and misfolded mSOD1. This is supported by the increased COMMD1 binding to mSOD1 compared to SOD1 WT (Figure 1), and its co-localization with mSOD1 aggregates (Figure 4). Furthermore, COMMD1 binding to mSOD1 is lost upon substitution of SOD1 cysteine 6 into a serine. Similar results were seen for its binding to SOD1 WT suggesting that SOD1 C6 is essential for COMMD1 interaction with both WT and mutant SOD1. This further implies that COMMD1 interacts with monomeric or small oligomeric mSOD1 proteins as its interaction is lost when the mSOD1 protein is unable to form intermolecular disulfide bonds. Since SOD1 C6 is also important for the formation of detergent-insoluble mSOD1 aggregates (Figure 5 and [34], [47], [48]), we cannot conclude whether the loss-of-aggregation properties of the double mSOD1 C6S/G93A affects the interaction between COMMD1 and mSOD1, or C6S substitution itself.

Yet, because of the irrelevance of cellular copper levels for the binding of COMMD1 to mSOD1, which is in contrast to the COMMD1- SOD1 WT interaction [14], the biochemical nature of the COMMD1 interaction with WT and mutant SOD1 is suggested to be distinct. This is underscored by mSOD1 G85R, a mutation located within the metal-binding region of SOD1 [49]–[51]. This mutation results in a lower copper affinity [52], but still markedly increased the interaction between SOD1 and COMMD1 (Figure 1). The finding that the copper chaperone for SOD1 (CCS), which delivers copper to SOD1, is suggested not to be required for mSOD1 aggregation in mice [38], whereas it is essential for COMMD1-SOD1 WT binding [14], further supports the different natures of COMMD1 binding to SOD1 WT and mutant. ALS-linked mutations in SOD1 result of exposure of more hydrophobic surfaces in the mSOD1 proteins, which enhances the propensity for non-native interactions with itself and other proteins and subsequent self- and co-aggregation [53], [54], and this may be the underlying mechanism driving the increased interaction of COMMD1 with mSOD1. Subsequently, the formation of this protein-protein complex intensifies the aggregation of proteins, which appears to be initiated by mSOD1 (Figure 4). Of note, COMMD1 has also the tendency to form HMW complexes with itself and its substrates (data not shown, and [6], [55], [56]); in this case, these are most likely exacerbated by mSOD1.

Previously, we demonstrated that binding of COMMD1 to SOD1 WT reduces the level of SOD1 WT dimers [14], and dimer dissociation is shown to initiate SOD1 aggregation *in vitro* [57], [58]. Additionally, co-expression of SOD1 WT exacerbates the disease phenotypes of mSOD1 mice (i.e. A4V, G85R, G93A)[59], [60], while mice overexpressing SOD1 WT alone are not characterized by paralysis or premature death [59]. Therefore, despite the fact that a mutation in *SOD1* enhances its interaction with COMMD1, we cannot rule out that the COMMD1-mediated reduction in endogenous SOD1 dimers and thus the expansion in monomeric WT species cause the increased formation of mSOD1 aggregates. Future structural and biochemical studies determining the effect of COMMD1 on mSOD1 aggregation in the absence of endogenous SOD1 are needed to provide further insight into the mechanism by which both COMMD1 and SOD1 WT affect mSOD1 aggregation.

Interestingly, our findings show that COMMD1 modifies misfolded protein aggregation in a client-specific manner. These data support the idea that the biochemical and biophysical properties of the various aggregation-prone proteins studied are substantially different, which may explain the client-specific actions of COMMD1. Unlike pathogenic Htt, mSOD1 proteins initially do not form dense, ordered amyloid-like fibrils but rather arrange a distinct amorphous, porous aggregate structure [61]. Also, the different misfolded proteins are accumulating in various cellular compartments when expressed in yeast or mammalian cells [62], [63]. Further support for this can be derived from the differential ability of the multiple heat shock proteins (HSPs) to reduce aggregation, which is highly depended on the aggregation-inducing client, and from unbiased screens for modifiers of the aggregate formation of different aggregation-prone proteins that have shown only limited overlap [63].

The client-specific effects of COMMD1 was recently also demonstrated in other studies. For example, we demonstrated that COMMD1 enhances the protein degradation of ATP7B [3], whereas COMMD1 improves the stability and copper transporting activity of misfolded ATP7A mutants [14]. Based on this knowledge, we suggest that COMMD1 improves the folding of mutant Parkin C289G and thereby reduces its aggregation. Alternatively, and not mutually exclusive, the aggregates of the mutant Parkin may be related to a dominant negative effect on the E3 ligase function of the protein, which may be prevented by COMMD1 binding, resulting in effective degradation of Parkin clients. This is further supported by recent findings demonstrating that COMMD1 activates the Cullin-RING ligases (CRLs) by antagonizing CAND1 (Cullin-associated Nedd8-dissociated protein 1, inhibitor that promotes dissociation of substrate-receptor components from CRLs) [64]. In this way, COMMD1 could promote the poly-ubiquitination of its substrates leading to their enhanced degradation. It might be possible that COMMD1 enhances the proteolysis of mutant Parkin C289G, similar as was found for ATP7B and HIF-1α [3], [5]. However, in contrast to its potential mode of action on Parkin proteins, no effect of COMMD1 on the protein stability of either SOD1 WT or mutants was observed ([14] and data not shown), which is consistent with the COMMD1-mediated induction in mSOD1 aggregation. It is clear that more mechanistic and physiological work will be needed to clarify both the mode of action as well as substrate-specificity of COMMD1 in the context of protein misfolding and aggregation, and what the consequences of these actions are for the toxicity related to these different aggregates.

Altogether, our and other studies are suggestive for COMMD1 to be a scaffold-like protein that acts at a platform to modify the characteristics and function of different proteins involved in various cellular processes. From our data, it can be inferred that COMMD1 should be included in the class of molecular chaperones that modulate cellular proteostasis, and thus could be a relevant player in the pathology of a range of protein-aggregation diseases.

## Appendix

### GST pull down assays using magnetic beads

Precipitation of GST-tagged proteins by means of glutathione magnetic beads was performed according to manufacturer’s protocol. In short, transient transfected cells were rinsed once with PBS prior to lysis in lysisbuffer A (25 mM HEPES; pH 7.9, 100 mM NaCl, 1 mM NaEDTA, 1% Triton X-100, 10% Glycerol) supplemented with 1 mM Na_3_VO_4_, 1 mM PMSF, 10 mM DTT and protease inhibitors (Complete; Roche). For lysis of copper-treated cells, lysis buffer A was supplemented with 1 mM CuCl_2_. Protein concentrations were determined by Bio-Rad (Bradford) Protein Assay (Bio-Rad Laboratories Inc., Hercules, CA, USA). In all interaction studies, equal amounts of proteins in lysisbuffer A were used for precipitation. GSH-magnetic beads (Pierce biotechnology, Rockford, IL, USA) were washed three times with cold binding/wash buffer (125 mM Tris-HCL, 300 mM NaCl, pH 8.0) using a magnetic stand prior to 1.5 h incubation with lysates at 4°C on a rotating platform. Subsequently, beads were collected using a magnetic stand, supernatant was removed, and beads were washed 4 times with binding/wash buffer. Elution was accomplished by two rounds of incubation of beads with 50 mM reduced L-glutathione (Sigma Aldrich) in binding/wash buffer, pH 8.0 for 10 min. Protein lysates were boiled at 95°C prior to gel loading, and SDS–PAGE was followed by proteins transfer onto nitrocellulose membranes (Schleicher & Schuell,‘s-Hertogenbosch, the Netherlands) for immunoblot analysis. Input samples represent approximately 1% of protein amounts used for GST-precipitation.

## Supporting Information

Figure S1 **Enhanced interaction of COMMD1 with ALS-associated SOD1 mutant proteins relative to SOD1 wild-type. A.** HEK293T cells were transient transfected with empty vector (EV), HA-COMMD1 alone or in combination with SOD1-GST constructs (WT, G85R, D90A, G93A). GST-proteins were precipitated by means of GSH-magnetic beads prior to detection of their interaction with COMMD1 as visualized by immunoblotting for HA-COMMD1 (GST PD; upper panel). 30 μg of protein lysates were used for detection of WT and mutant SOD1-GST and HA-COMMD1 in total cell lysates (Input; lower panel) using antibodies directed against the HA- or GST-fusion proteins. Graph represents densitometric quantification of interaction strength between COMMD1 and SOD1 WT versus SOD1 mutants (G85R, D90A, G93A; GST PD), normalized for total SOD1 expression (input). Binding of COMMD1 to SOD1 WT was set at 1. **B.** HEK293T cells were transient transfected with GST, COMMD1-GST alone or in combination with SOD1-Flag constructs (WT, G85R, D90A, G93A). GST-proteins were precipitated by means of GSH-magnetic beads prior to detection of their interaction with SOD1 as visualized by immunoblotting for SOD1-Flag (GST PD; upper panel). 30 μg of protein lysates were used for detection of GST, COMMD1-GST, and WT and mutant SOD1-Flag in total cell lysates (Input; lower panel) using antibodies directed against the GST- or Flag-fusion proteins. Graph represents densitometric quantification of interaction strength between COMMD1 and SOD1 WT versus SOD1 mutants (G85R, D90A, G93A; GST PD), normalized for total SOD1 expression (input). Binding of COMMD1 to SOD1 WT was set at 1. **C.** HEK293T cells were transient transfected with HA-COMMD1 in combination with SOD1-GST constructs (WT, G85R, D90A, G93A). Cells were incubated overnight under basal conditions (–) or with 150 μM CuCl_2,_ lysed, and GST fusion proteins were precipitated by means of GSH-magnetic beads prior to detection of their interaction with COMMD1 as visualized by immunoblotting for HA-COMMD1 (GST PD; upper panel). 30 μg of protein lysates were used for detection of SOD1-GST WT and mutants and HA-COMMD1 in total cell lysates (Input; lower panel) using antibodies directed against the HA- or GST-tags.
(PDF)

Figure S2 **Enhanced interaction of COMMD1 with Parkin C289G mutant proteins relative to wild-type Parkin.** HEK293T cells were transient transfected with GST or COMMD1-GST alone in combination with Flag-Parkin constructs (WT and C289G). Cells were lysed, and GST fusion proteins were precipitated as described under Figure S1A. Immunoblotting was performed using indicated antibodies.
(PDF)

Table S1 **Oligonucleotide primers used in this study.**
(DOCX)
