# Supplementary figures and images for: The Copper Metabolism MURR1 Domain Protein 1 (COMMD1) Modulates the Aggregation of Misfolded Protein Species in a Client-Specific Manner
Source: PLoS One. 2014 Apr 1;9(4):e92408. doi: 10.1371/journal.pone.0092408 (PMC3972230; doi:10.1371/journal.pone.0092408)

Figure S1.

A.

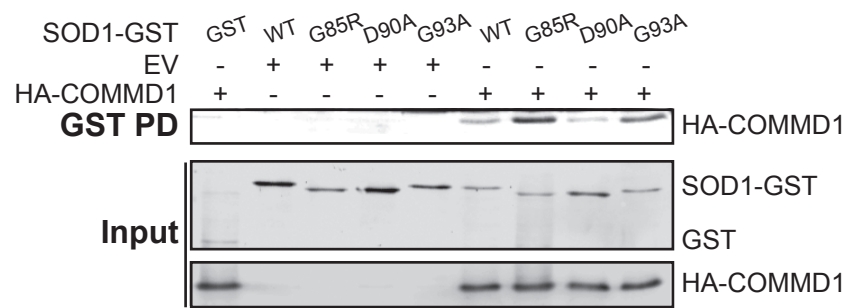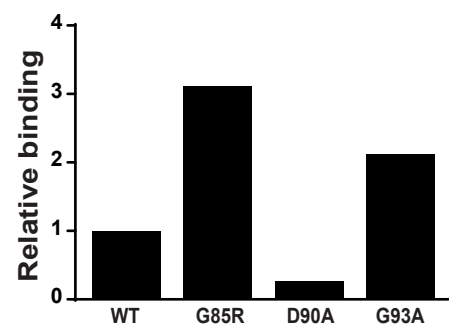

B.

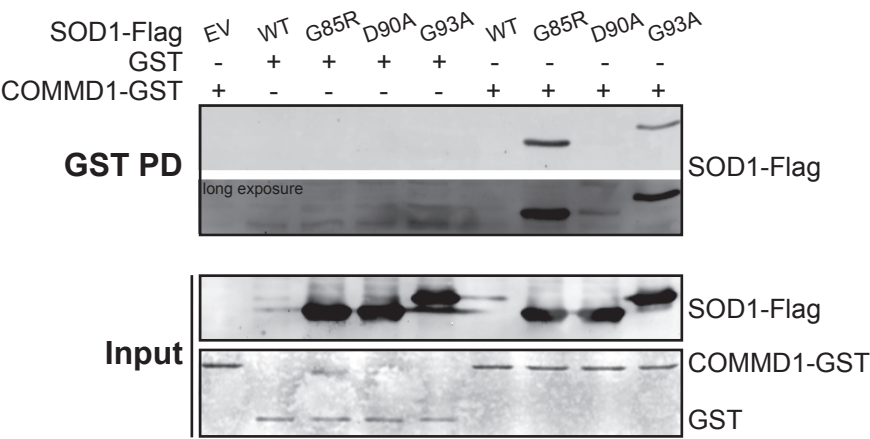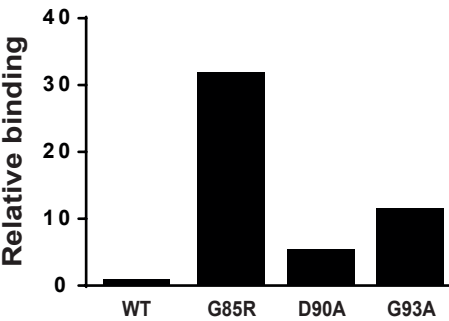

C.

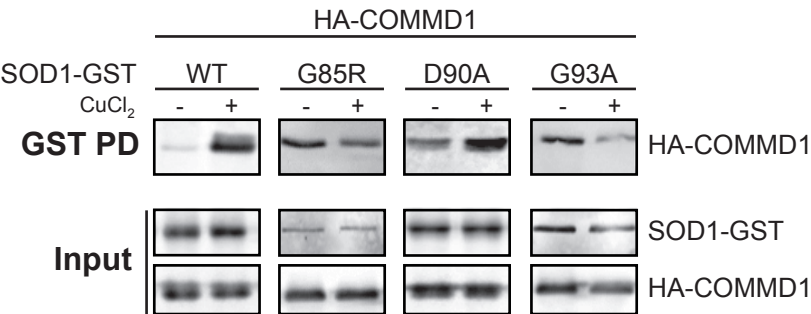

Supplement: Figure S1 — Enhanced interaction of COMMD1 with ALS-associated SOD1 mutant proteins relative to SOD1 wild-type. A. HEK293T cells were transient transfected with empty vector (EV), HA-COMMD1 alone or in combination with SOD1-GST constructs (WT, G85R, D90A, G93A). GST-proteins were precipitated by means of GSH-magnetic beads prior to detection of their interaction with COMMD1 as visualized by immunoblotting for HA-COMMD1 (GST PD; upper panel). 30 μg of protein lysates were used for detection of WT and mutant SOD1-GST and HA-COMMD1 in total cell lysates (Input; lower panel) using antibodies directed against the HA- or GST-fusion proteins. Graph represents densitometric quantification of interaction strength between COMMD1 and SOD1 WT versus SOD1 mutants (G85R, D90A, G93A; GST PD), normalized for total SOD1 expression (input). Binding of COMMD1 to SOD1 WT was set at 1. B. HEK293T cells were transient transfected with GST, COMMD1-GST alone or in combination with SOD1-Flag constructs (WT, G85R, D90A, G93A). GST-proteins were precipitated by means of GSH-magnetic beads prior to detection of their interaction with SOD1 as visualized by immunoblotting for SOD1-Flag (GST PD; upper panel). 30 μg of protein lysates were used for detection of GST, COMMD1-GST, and WT and mutant SOD1-Flag in total cell lysates (Input; lower panel) using antibodies directed against the GST- or Flag-fusion proteins. Graph represents densitometric quantification of interaction strength between COMMD1 and SOD1 WT versus SOD1 mutants (G85R, D90A, G93A; GST PD), normalized for total SOD1 expression (input). Binding of COMMD1 to SOD1 WT was set at 1. C. HEK293T cells were transient transfected with HA-COMMD1 in combination with SOD1-GST constructs (WT, G85R, D90A, G93A). Cells were incubated overnight under basal conditions (–) or with 150 μM CuCl2, lysed, and GST fusion proteins were precipitated by means of GSH-magnetic beads prior to detection of their interaction with COMMD1 as visualized by immunoblotting for H [file pone.0092408.s001.pdf]

Figure S2.

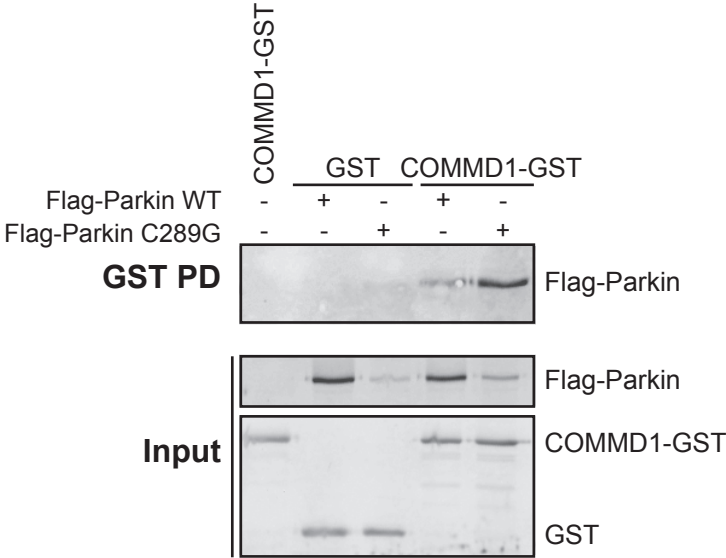

Supplement: Figure S2 — Enhanced interaction of COMMD1 with Parkin C289G mutant proteins relative to wild-type Parkin. HEK293T cells were transient transfected with GST or COMMD1-GST alone in combination with Flag-Parkin constructs (WT and C289G). Cells were lysed, and GST fusion proteins were precipitated as described under Figure S1A. Immunoblotting was performed using indicated antibodies. (PDF) [file pone.0092408.s002.pdf]
