# Supplementary material for: The Copper Metabolism MURR1 Domain Protein 1 (COMMD1) Modulates the Aggregation of Misfolded Protein Species in a Client-Specific Manner
Source: PLoS One. 2014 Apr 1;9(4):e92408. doi: 10.1371/journal.pone.0092408 (PMC3972230; doi:10.1371/journal.pone.0092408)
Supplement: Table S1 — Oligonucleotide primers used in this study. (DOCX) [file pone.0092408.s003.docx]

**Table S1. Oligonucleotide primers used in this study**

| **SOD1 mutation** | **Forward primer sequence (5’-> 3’)** | **Reverse primer sequence (5’-> 3’)** |
| --- | --- | --- |
| **A4V** | atggcgacgaaggtcgtgtgcgtgctgaagg | ccttcagcacgcacacgaccttcgtcgccat |
| **G37R** | gcattaaaagactgactg | cagtcagtcttttaatgc |
| **G85R** | gttggagacttgcgcaatgtgac | gtcacattgcgcaagtctccaac |
| **D90A** | caatgtgactgctgccaaagatggtgtgg | ccacaccatctttggcagcagtcacattg |
| **G93A** | gacaaagatgctgtggccgatgtg | cacatcggccacacgcatctttgtc |
| **E100G** | gtgtctattggagattctgtg | cacagaatctccaatagacac |
